# Membranous Replication Factories Induced by Plus-Strand RNA Viruses

**DOI:** 10.3390/v6072826

**Published:** 2014-07-22

**Authors:** Inés Romero-Brey, Ralf Bartenschlager

**Affiliations:** Department of Infectious Diseases, Molecular Virology, Heidelberg University, Im Neuenheimer Feld 345, 69120 Heidelberg, Germany

**Keywords:** membrane rearrangements, electron microscopy, electron tomography, ultrastructure, double-membrane vesicles, membranous replication factories, hepatitis C virus, flaviviruses, picornaviruses, coronaviruses

## Abstract

In this review, we summarize the current knowledge about the membranous replication factories of members of plus-strand (+) RNA viruses. We discuss primarily the architecture of these complex membrane rearrangements, because this topic emerged in the last few years as electron tomography has become more widely available. A general denominator is that two “morphotypes” of membrane alterations can be found that are exemplified by flaviviruses and hepaciviruses: membrane invaginations towards the lumen of the endoplasmatic reticulum (ER) and double membrane vesicles, representing extrusions also originating from the ER, respectively. We hypothesize that either morphotype might reflect common pathways and principles that are used by these viruses to form their membranous replication compartments.

## 1. The Family Flaviviridae

**Figure 1:**
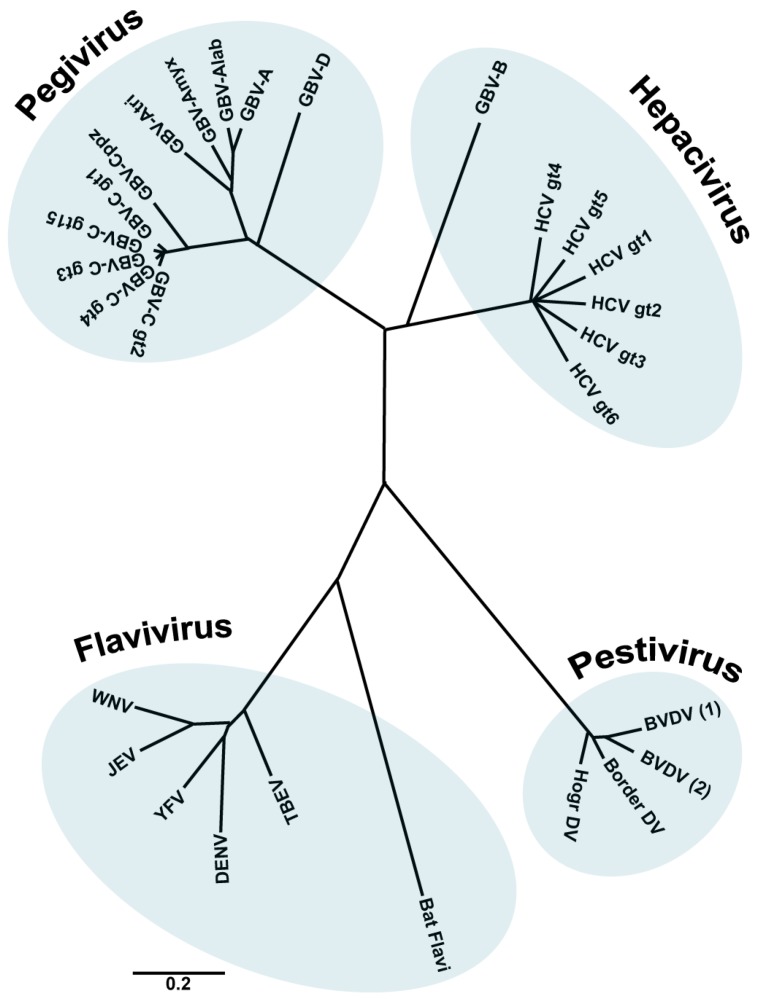
The family *Flaviviridae*. Phylogenetic tree based on the analysis (neighbour joining) of aligned conserved motifs of the RNA dependent RNA polymerase (RdRp). Shown are selected members of the family. A distance scale corresponding to amino acid substitutions per position is shown. (Figure adapted from [1], with permission).

Members of the family *Flaviviridae* are enveloped viruses with a single stranded RNA genome of positive polarity. This family contains four different genera: *Hepacivirus* (from the Greek *hepar*, *hepatos*, “liver”), *Flavivirus* (from the Latin *flavus*, “yellow”), *Pestivirus* (from the Latin *pestis*, “plague”) and the recently included genus *Pegivirus* [1,2] (Figure 1).

Hepatitis C Virus (HCV) is the prototype species of the genus *Hepacivirus*. It was first discovered by Choo *et al.* [3] in the serum and tissues of a chimpanzee experimentally inoculated with serum from an individual with chronic, non-A, non-B hepatitis. This virus was associated with a mild form of chronic hepatitis frequently observed in recipients of blood transfusions [4] and was called HCV. A second species within the genus *Hepacivirus* is GBV-B which was first identified in tamarins that developed hepatitis following inoculation with the serum from a surgeon (initials G.B.) with acute hepatitis. Additional GB-like viruses were discovered later on and have been assigned to the new genus *Pegivirus* (an acronym derived from *pe*, persistent; *g*, GB or G) within the family *Flaviviridae* [1].

The genus *Flavivirus* comprises 53 species and represents the largest genus within the family *Flaviviridae.* Many of these viruses are transmitted by arthropods and therefore called ARBO viruses (**ar**thropod-**bo**rne). Flaviviruses such as Dengue Virus (DENV), Japanese Encephalitis Virus (JEV), West Nile Virus (WNV) or Yellow Fever Virus (YFV) cause a variety of diseases, including fever, encephalitis and hemorrhage [5,6].

Pestiviruses are important animal pathogens causing major losses in stock farming. The genus comprises the species Bovine Viral Diarrhea Virus 1 and 2 (BVDV-1/-2), Border Disease Virus (BDV), Classical Swine Fever Virus (CSFV), the tentative virus species “Giraffe”, as well as several unassigned viruses [7]. Within the family *Flaviviridae*, pestiviruses show greater similarity in genome structure and mechanism of translation initiation to the hepaciviruses than to flaviviruses. Pestivirus infections can be subclinical or induce a range of clinical conditions, including acute diarrhea, acute hemorrhagic syndrome, acute fatal disease, and a wasting disease [5].

These viruses have in common a single strand RNA genome containing one long open reading frame flanked by 5’- and 3’-untranslated regions (UTRs) [5]. Viral proteins are produced by cleavage of a single polyprotein that has a length of about 3000 amino acid residues. The N-terminal half of the polyprotein contains the structural proteins forming the virus particle, while the C-terminal part contains the nonstructural proteins (NS) involved in synthesis of the viral RNA genome. The NS proteins comprise the enzymatic components of the RNA replicase, including a RNA helicase and a RNA-dependent RNA polymerase (RdRp) [8].

Binding and uptake of viruses belonging to the *Flaviviridae* family into their host cells occur by receptor-mediated endocytosis involving interactions between cellular receptors and viral envelope proteins. Upon uncoating, the (+) RNA genome is released into the cytoplasm, where it serves as messenger RNA (mRNA) for translation of all viral proteins, as template for RNA replication to produce new (−) RNA copies and as genetic material packaged into new virus particles. During RNA replication, the (+) RNA genome is converted into a (−) RNA copy that serves as template for production of new (+) RNA genomes. Most of these steps occur in close association with intracellular membranes that are rearranged upon viral infection giving rise to complex membranous replication factories. These rearrangements correspond to invaginations or protrusions of membranes that are frequently derived from the endoplasmic reticulum (ER). In this review, we summarize our current knowledge about the 3D architecture of these complex membrane rearrangements, focusing on members of the family *Flaviviridae* that generate either morphotype of replication compartment.

## 2. Architecture and Properties of the Replication Factories of Members of the *Flaviviridae* Family

### 2.1. Flaviviruses

**Figure 2:**
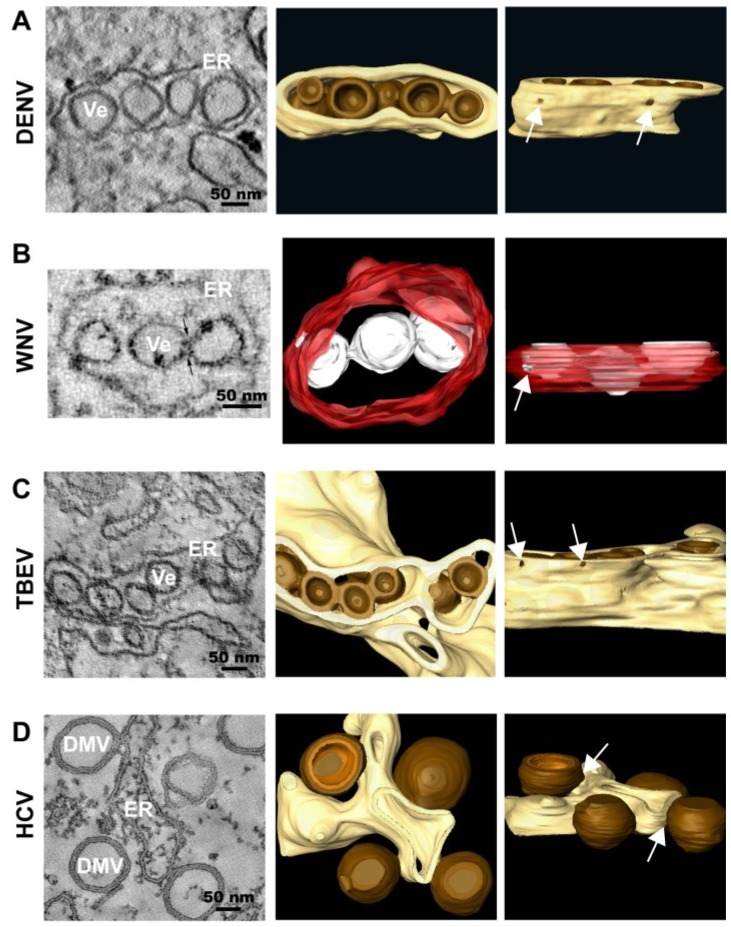
Representative images of membrane rearrangements induced by different members of the family *Flaviviridae*. (**A**) Dengue Virus (DENV); (**B**) West Nile Virus (WNV); (**C**) Tick-borne Encephalitis Virus (TBEV); (**D**) Hepatitis C Virus (HCV). Slices through tomograms of infected cells (**on the left**) and 3D top and lateral (90° rotation) views of the same tomograms (**on the right**) are depicted, showing the characteristic virus-induced structures. The replication vesicles (Ve) of DENV, WNV and TBEV (genus *Flavivirus*) correspond to invaginations of ER membranes that remain connected to the cytosol via 10 nm-pores (highlighted with white arrows in the 3D lateral views), forming vesicle packets (VPs). The replication factory of HCV (genus *Hepacivirus*) is primarily composed of double membrane vesicles (DMVs) that seem to be formed asER protusions connected to ER membranes via neck-like structures (highlighted with white arrows in the 3D lateral view). The ER is shown in yellow (DENV, TBEV and HCV) or in red (WNV) and the replication organelles in brown (DENV, TBEV and HCV) or in white (WNV). The outer and inner membranes of DMVs are depicted in different shades of brown (outer membrane in dark brown and inner membrane in light brown). Figure 2B is reproduced with permission from [20].

The first publications on DENV-induced membrane modifications in mammalian cells (marked cytoplasmic vacuolization and accumulation of virions in crystalloid arrays surrounded by membranes) date from the seventies [9,10]. Several years later, studies on DENV-infected mosquito cells were published in which the visualization of virions was reported [11,12]. In 1997, Grief and coworkers described DENV-induced membrane alterations of various morphologies, including convoluted membranes (CMs) and vesicle packets (VPs) [13]. Subsequent immuno-EM studies indicated that VPs and CMs may represent the site of DENV replication and RNA translation/polyprotein processing, respectively [14]. The most complete characterization of DENV-induced intracellular membrane rearrangements elucidated their 3D architecture as well as their spatial connection with viral assembly sites [15]. TEM of resin-embedded infected cells revealed a complex collection of convoluted and vesicular structures, including CMs that were usually surrounded by multiple vesicles, often appearing as longitudinal vesicle arrays. By using electron tomography (ET), the latter were found to correspond to ER tubules containing 80–90 nm single-membrane vesicles (Ve) that result from the invagination of the ER membrane into the ER lumen. By conventional EM, these vesicles appeared as double membrane vesicles, likely corresponding to the VPs described earlier [13]. Immuno-EM confirmed that the vesicles visible in resin-embedded cells were induced by DENV infection and contained all NS proteins. However, only NS3 was detectable within the CMs, which could be due to lower affinity of the antibodies or poor accessibility of the other NS proteins in the CMs. Double-stranded RNA (dsRNA) detected by immunostaining appeared as discrete electron-dense structures inside or on the cytosolic surface of a subset of vesicles, suggesting that dsRNA might be present only in some of the vesicles at a given time point. Furthermore, the vesicles contain rather uniform pores of ~10 nm diameter towards the cytosol (Figure 2A). Thus, both the topology of the vesicles and the immunolabeling results support the idea that the vesicles might be the site of RNA replication. Moreover, these results showed that replication factories are a continuous membrane network that provides a platform for the transport of viral proteins and genomes between sites of RNA replication, ribosome-containing compartments (RNA translation) and virus assembly sites. In fact, virus budding sites were found in close proximity to the pores of the replication vesicles. This topological link may ensure efficient production and delivery of viral RNA for the assembly of infectious virus progeny. Consistent with these findings, a very recent publication using ET showed that these virally modified structures were also observed in DENV-infected mosquito cells, with one exception: CMs were absent from DENV-infected C6/36 mosquito cells [16]. In addition, after multiple rounds of virus replication, tubular structures were also observed in the vicinity of VPs. These structures might represent a hallmark of chronically infected insect cells, since these structures are also induced by TBEV in tick cells (see below).

The first reports on WNV-infected cells described the visualization of virions [17]. An extensive characterization of Kunjin Virus—the Australian variant of WNV (WNV_KUN_)—infected cells has been carried out more recently [18,19,20]. Three well-defined structures were found, corresponding to large CMs, paracrystalline arrays (PCs) and VPs that appeared as membrane sacs containing small vesicles (Ve) [18,21]. Based on immunolocalization studies, a distinct redistribution of the *trans*-Golgi network (TGN) and colocalization of TGN markers with dsRNA has been observed, suggesting that the replication factories of WNV_KUN_ were derived from the TGN [22]. Three-dimensional reconstructions of the WNV_KUN_ replication sites revealed an intimate association of the rough ER (rER) with the bounding membrane of the VPs [20] (Figure 2B), resembling the vesicles observed in DENV-infected cells. These results argue for an additional role of the rER in the formation of the WNV_KUN_ replication factories. Similar to DENV, individual necks were observed in the vesicles as well as the majority of the viral RNA, as detected by immunolabeling with a dsRNA-specific antibody, resided within these vesicles [18,20,22,23,24]. In most cases, viral RNA spanned the breadth of the vesicles and was juxtaposed to the necks open to the cytoplasm [20].

In cells infected with TBEV, one of the most important tick-transmitted viruses in Europe and Asia, virus particles and membrane-connected vesicles were also observed inside the ER [25], similar to what was described for DENV and WNV_KUN_. The viral dsRNA was only detected inside the vesicular structures within rER, suggesting that TBEV rearranges internal cell membranes to generate a compartment that protects viral RNA from detection by cytoplasmic pathogen recognition receptors (PRRs) [26,27,28]. This localization of dsRNA might suffice to delay the onset of the IFN response [25]. For TBEV [25] and WNV_KUN_ [19] it was shown that treatment with brefeldin A (BFA), a drug which disrupts the Golgi apparatus, did not interfere with viral replication. However, this treatment rendered WNV_KUN_ sensitive to the antiviral action of the IFN-induced protein MxA, indicating that BFA might have disrupted the membranous WNV_KUN_ replication compartments, thus leading to exposure of dsRNA and its detection by PRRs. In contrast, treatment of TBEV-infected cells with BFA neither affected viral replication, nor the level of IFN production. These findings indicate that TBEV dsRNA might be stored inside BFA-resistant membrane vesicles that robustly protect the viral RNA from recognition by cellular sensors.

Vector-borne flaviviruses like DENV and TBEV must replicate in both mammalian and arthropod cells. A few comparative studies have been published describing virus-induced structures such as cytoplasmic membrane proliferations and vesicle formation, also in insect cells [11,16,21,29,30,]. A detailed comparative ultrastructural analysis of TBEV-induced modifications revealed that the extent of membrane expansion and the abundance of vesicles were lower in insect cells [31]. Single-membrane vesicles, ranging in diameter from 60–100 nm were frequently found within proliferated ER areas, often occurring in large groups contained within ER cisternae. Pore-like openings connected these vesicles to the cytoplasm and to other vesicles. Apart from these vesicles, in tick-infected cells elongated vesicles or tubules were found that were much more prevalent in persistently than in acutely infected cells. These tubules were only occasionally noted in infected mammalian cells, similar to what was found with DENV-infected cells [15]. The tubular structures had a cross-sectional diameter of 60–100 nm, similar to the one of vesicles, reached up to 800 nm in length, were closed at the ends and often arranged in fascicle-like bundles, shrouded with the ER membrane. However, no pores between the tubules or towards the cytoplasm were observed [31]. The function of these tubules is unclear and it is not known whether they represent *bona fide* features of replication factories, aberrant structures as a result of incorrect membrane remodeling, or the result of a cellular process to restrict infection [31]. In any case, the tubules might be a feature of persistent infection, eventually linked to the high number of defective virus particles, because the lack of pores could prevent proper replication or packaging of the viral genome [32,33,34]. Further studies are required to shed light on the biogenesis and biological significance of these membranous tubular structures.

A recent study identified 80 nm-diameter vesicles within the ER lumen of TBEV-infected BHK-21 cells and in cells transfected with a TBEV replicon [35]. ET revealed that these vesicles are invaginations of the ER within a highly organized network of interconnected membranes with half of vesicles containing pore-like connections to the cytoplasm (Figure 2C). However, no pore-like openings were observed between adjacent/neighboring vesicles, in contrast to what has been described for cells infected with Langat Virus (LGTV), a naturally attenuated tick-borne flavivirus [31] or in WNV_KUN_-infected cells [20]. Interestingly, in TBEV replicon cells, the number of pore-containing vesicles was slightly larger (~75%) and they were found in much more fragmented ER tubules as compared to TBEV-infected cells. However, despite more extensive ER rearrangements in replicon cells, they contained fewer vesicles, consistent with the lower level of viral replication [36,37,38].

Conventional EM analysis of neurons infected with Murray Valley Encephalitis Virus (MVEV) revealed several ultrastructural features, including proliferation of ER and Golgi complex membranes as well as the appearance of membrane-bound spherical vesicles (75–145 nm diameter) [39], similar to those observed for the related flaviviruses Japanese Encephalitis Virus (JEV) [40,41] and St. Louis Encephalitis Virus (SLEV) [42]. In the latter case, cylindrical membranous structures (or tubules) were also observed [43]. The presence of vesicles was also detected in monkey liver cells infected with Yellow Fever Virus (YFV) [44]. These findings indicate that all members of the genus *Flavivirus* utilize the ER as a source of membranes for the formation of their replication factories, whereas assembly of new virions seems to occur at ER sacs in close proximity to the replication sites [15,35], thus creating an optimized membranous environment to support efficient viral replication and assembly. Maturation of the newly synthesized virions takes place in the Golgi apparatus, where flavirirus virions are often observed [15].

### 2.2. Hepaciviruses

In stark contrast to flaviviruses, HCV, the prototype of the genus *Hepacivirus*, provokes an alternative rearrangement of intracellular membranes, originally designated “membranous web” (MW). This term referred to compact vesicle accumulations embedded into a membranous matrix [45] as detected in cells inducibly expressing the HCV polyprotein. By using different EM methods, we and others have recently found that the MW is primarily composed of double membrane vesicles (DMVs) [46,47,48]. The fact that the kinetics of their appearance correlates with HCV replication suggests that these structures play an important role for viral RNA amplification [48]. Indeed, immunolabeling of purified DMVs revealed an enrichment for viral proteins as well as dsRNA [46,49]. Importantly, DMVs contain enzymatically active viral replicase [49] and they originate from ER membranes, similar to what has been found for other members of the family *Flaviviridae*. ET analysis showed that most of the DMVs remain connected to the ER via their outer membrane [48] (Figure 2D). Although DMVs are primarily closed structures, ~10% of them have an opening towards the cytosol. Late in infection, multi-membrane vesicles (MMVs) with an average diameter of 390 nm are generated, likely originating from DMVs by secondary enwrapping events [48].

By using Huh7.5 cells infected with the highly replicative HCV strain JFH-1, Ferraris and coworkers observed three different types of membrane alterations: vesicles in clusters (ViCs), contiguous vesicles (CVs) and DMVs [47]. The ViCs were small single-membrane vesicles of variable size (100–200 nm), grouped together in well-delimited areas. Most of them had an internal invagination. The CVs were small single-membrane vesicles, present in large numbers and widely distributed throughout the cytoplasm, with a more homogeneous size (around 100 nm). They were tightly associated to each other and tended to form a collar around lipid droplets (LDs). DMVs were heterogeneous in size (150–1000 nm) and had a thick, electron-dense membrane consisting of two closely apposed membranes. The increase of CVs’ number correlated with an increase of intracellular HCV RNA levels, arguing for a possible role of CVs in the early stages of viral replication. The presence of NS5A in CVs, as demonstrated by immunogold staining, is consistent with this hypothesis. Alternatively, CVs might constitute the membranous platform for viral assembly. In fact, the core protein is present in these structures (16%) as well as on the LD surface (81%). However, so far visualization of virus particles in infected cells has not been possible, making this hypothesis difficult to prove. While most of the dsRNA signal was located within DMVs or at DMV membranes, ViCs were free of viral components and RNA and these structures as well as CVs were very rarely observed in cells with a subgenomic JFH-1 replicon [46] or absent in cells infected with a JFH-1 variant designated Jc1 [48]. The first 3D reconstruction of a complete HCV-infected cell revealed that all these three membrane structures were tightly connected and closely associated with LD clusters [47].

Taken together, these findings indicate a fundamental role of DMVs in HCV replication. An in-depth comparison of the study by Ferraris and coworkers [47] and our publication [48] suggests that CVs might be also DMVs for several reasons: first, CVs have electron dense tightly apposed membranes; second, by using correlative light and electron microscopy, we also detected DMV accumulations around LDs, reminiscent of the CVs described by Ferraris and coworkers [47]; third, taking into consideration the density of content and morphology, some of the structures described as DMVs by Ferraris and colleagues might correspond to MMVs according to our nomenclature. This might account for the differences in size between the DMVs reported in both studies (up to 1000 nm *versus* 150 nm, respectively). Alternatively, the difference might be due to the use of distinct virus strains (JFH-1 and Jc1) that differ in their capacity to produce infectious virus particles by ~3 orders of magnitude [50], which might also explain the presence of ViCs only in JFH-1 infected cells.

### 2.3. Pestiviruses

Much less about membranous replication factories is known for pestiviruses. TEM-based studies from the times in which the genus *Pestivirus* was still belonging to the family *Togaviridae* reported that pestivirus-infected cells exhibited ultrastructural modifications of rER and contained small numbers of virus-like particles (VLPs) [51,52]. Gray and Nettleton (1987) reported that Border Disease Virus (BDV)-infected cells contained several profiles of ER and many dense lamellar bodies, which when transversely sectioned appeared as multiple rows of tubules, 33 nm in diameter [52]. These lamellae were often found in association with rER and in one occasion VLPs appeared to be budding within them. Bovine Viral Diarrhea Virus (BVDV)-infected cells contained rER modified into tubules, in which electron-dense VLPs were present. More recent studies on BVDV-infected cells revealed cytoplasmic vacuolization and VLPs in dilated ER cisternae [53,54]. In addition, membrane structures consisting of vesicles of various sizes enclosed in much larger vesicles have been reported [55]. These structures that morphologically resemble multivesicular bodies (MVBs) are distinct from the HCV-induced membranous web and more reminiscent of the flavivirus-induced VPs.

Studies on the morphogenesis of pestiviral particles were hampered by a low rate of virion production. In a recent study, Schmeiser and colleagues have overcome this problem by using high multiplicity of infection in MDBK cells with a distinct virus strain, the Giraffe-1 strain [56]. Obtained results define the ER as the site of pestivirus particle assembly, where budding of virions was observed. Virus particles were also found inside the lumen of the Golgi and in vesicles associated with the Golgi compartment, suggesting that virus egress occurs via the conventional secretory pathway. Interestingly, replication kinetics of pestiviral RNA did not correlate with distinct membrane rearrangements and only slight dilatation of the ER lumen was noticed. The absence of significant membrane rearrangements argues for a major difference between pestiviruses and other members of the *Flaviviridae* family. Interestingly, the authors detected the capsid protein and dsRNA, the marker for viral replication intermediates, mainly in MVBs, indicating that pestiviruses are either using this compartment for replication or that viral RNA and proteins are transferred to this compartment for degradation. Similar assumptions have been made for HIV [57] and Marburg virus, a member of the *Filoviridae* family [58,59]. Alternatively, pestiviral RNA and protein in MVBs might be intermediates of the entry process, prior to fusion of the envelope with the endosomal membrane. Indeed, particles inside MVBs matching the morphological criteria of pestivirus virions were detected [56]. However, MVBs of non-infected cells also contain vesicles for lysosomal degradation termed intraluminal vesicles (ILVs) that display a very similar morphology to pestiviral virions. Thus, unambiguous discrimination between ILVs and pestivirus particles will require detailed immunolabeling approaches.

## 3. Architecture and Properties of the Replication Factories of Other (+) Strand RNA Viruses

### 3.1. Nodaviruses

**Figure 3:**
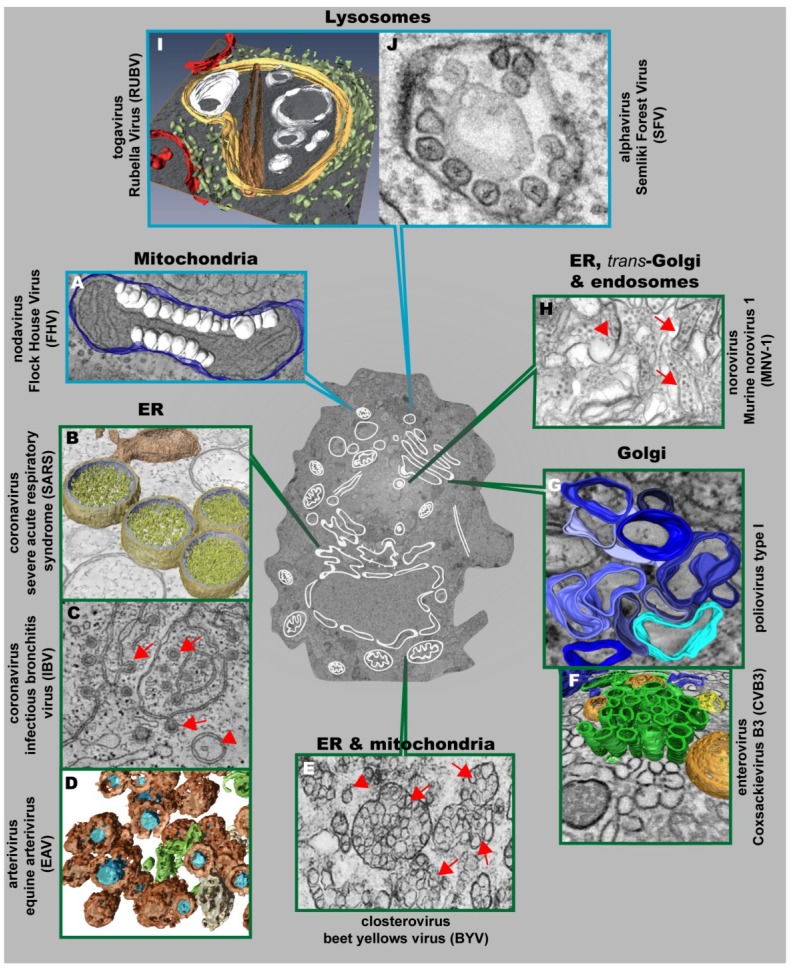
Positive strand RNA viruses usurp and modify cell membranes of different origins to replicate their genomes. (**A**) Mitochondrial membranes are targeted by Flock House Virus (FHV), which induces the formation of vesicles (white) at the outer mitochondrial membrane (blue); (**B**) 3D surface-rendered model of SARS-CoV–infected Vero E6 cells containing large double membrane vesicles (DMVs) (outer membrane, gold; inner membrane, silver) that remain connected to their source, the ER (in bronze); (**C**) Zippered ER, found in cells infected with the gammacoronavirus Infectious Bronchitis Virus (IBV), is connected to spherules (red arrows). DMVs (red arrowheads) are also found, but to a lesser extent; (**D**) Equine Arteriviruses (EAV) infection of HeLa cells results in the formation of DMVs (brown), depicting a core (blue) that is associated with the ER (beige) and close to ER tubules (green); (**E**) Closterovirus rearranges ER and mitochondrial membranes to form DMVs (red arrowheads) and vesicles packets (VPs) (red arrows); (**F**) Coxsackie B3 Virus (CVB3) usurps donor membranes most likely derived from the Golgi, appearing early in infection as single-membrane tubules (green), open (orange) and closed (yellow) DMVs and ER (blue); (**G**) 3D architecture of poliovirus membranous replication factories at intermediate stages of development, originating from *cis*-Golgi membranes. Single-membrane structures are depicted in different shades of blue. Note that these single membrane vesicles undergo secondary invaginations giving rise to DMVs at the late stages of infection; (**H**) Murine Norovirus 1 (MNV-1) infection results in the formation of vesiculated areas (VAs, red arrowheads) within aggregates of MNV-1 particles (red arrows). These VAs seem to originate from ER, *trans*-Golgi and endosomes; (**I**) 3D model of a cytopathic vacuole (CPV) found in Rubella Virus-infected cells. This CPV (yellow) is surrounded by the rER (light green) and contains a number of vacuoles, vesicles (white) and a rigid straight sheet (brown) that is connected with the periphery of the CPV. Mitochondria are depicted in red; (**J**) Typical cytoplasmic vacuoles induced by Semliki Forest Virus (SFV) in baby hamster kidney cells. Color code is as follows: blue-framed images depict single-membrane vesicle inducers; green-framed images correspond to double-membrane vesicle inducers. (The different parts were reproduced with permission: see acknowledgement section)

The first visualization of the 3D architecture of a (+) strand RNA virus replication factory was reported for Flock House Virus (FHV), a member of the family *Nodaviridae* [60]. This insect nodavirus induces the formation of invaginations at the outer mitochondrial membrane (OMM) with an average diameter of ~50 nm [60,61] (Figure 3A). The interior of these vesicles (called spherules) is connected to the cytoplasm by a necked channel of ~10 nm diameter, which is wide enough to allow import of ribonucleotides and export of synthesized RNA (diameter < 2 nm) [60]. Furthermore, metabolically labeled FHV RNA localized between inner and outer mitochondrial membranes inside these spherules, thus validating the spherules as *bona fide* FHV-induced compartments for viral RNA synthesis [60].

### 3.2. Nidovirales: Corona- and Arteriviruses

Conventional TEM analyses of coronavirus-infected cells identified large numbers of isolated DMVs [62]. At least in case of the Severe Acute Respiratory Syndrome (SARS)-Coronavirus, these DMVs are part of an elaborate reticulovesicular network (RVN) of modified ER that consists of convoluted membranes, numerous DMVs (diameter 200–300 nm) (Figure 3B) [63], and “vesicle packets” apparently arising from merging of DMVs. The CMs were most intensively immunolabeled for viral replicase subunits whereas DMVs labeled abundantly for dsRNA. While this result argues that DMVs might be the site of viral RNA synthesis, ET analyses failed to detect DMV connections to the cytoplasm to allow transport of nascent RNA. Instead, DMVs are connected to each other, to CMs and to the ER via their outer membranes.

Also, in case of another coronavirus, the Mouse Hepatitis Virus (MHV), ER membranes are thought to be the lipid donor of the membranous replication compartment [64,65]. Qualitative and quantitative analyses by (immuno)-electron microscopy of MHV-induced membrane rearrangements revealed the appearance, in strict order, of DMVs (diameter 200–350 nm), CMs, large virion-containing vacuoles, tubular bodies and cubic membrane structures [65].

The recently identified coronavirus Middle East Respiratory Syndrome Coronavirus (MERS-CoV) induces extensive membrane rearrangements in the perinuclear region, including the formation of DMVs and CMs [66]. The diameter of MERS-CoV induced DMVs ranged from 150–320 nm, comparable to the SARS-CoV induced DMVs. In addition, CMs were always surrounded by DMV clusters and were only observed in cells that appeared to be more advanced in infection. This observation strengthens the notion that DMV formation precedes the development of CMs, as postulated previously for SARS-CoV [63].

In addition to the betacoronaviruses (SARS-CoV, MHV and MERS-CoV), ER-derived DMVs with a diameter of ~200 nm have been observed in primary avian and mammalian cells infected with Infectious Bronchitis Virus (IBV), an important poultry pathogen belonging to the genus *Gammacoronavirus* [67]. However, the most striking structures induced by IBV are zippered ER membranes (Figure 3C). The zippered ER was associated to 60–80 nm diameter spherules, structures that are not present in cells infected with betacoronaviruses. ET showed that these IBV-induced spherules are tethered to the zippered ER and contain a 4.4 nm long channel connecting their interior to the cytoplasm of the cell, making them the ideal candidates for the site of IBV RNA synthesis.

ER membranes are also targeted by nidovirales that belong to the family *Arteriviridae*. Cells infected with the prototypic arterivirus, Equine Arterivirus (EAV), also contain DMVs associated to ER tubules. These DMVs are ~2–3 times smaller (~95 nm) as compared to coronaviruses [68]. A recent in-depth ultrastructural analysis revealed that the outer membranes of EAV-induced DMVs are interconnected with each other and with the ER (Figure 3D), thus forming a reticulovesicular network (RVN) resembling the one previously described for the distantly related SARS-CoV [69]. Despite significant morphological differences, a striking parallel between the two virus groups, and possibly all members of the order *Nidovirales*, is the accumulation of dsRNA, the presumed intermediate of viral RNA synthesis, in the DMV interior. Along these lines, DMVs visualized by means of electron spectroscopy imaging contained phosphorus in amounts corresponding on average to a few dozen copies of the EAV RNA genome. Like in SARS-coronavirus infected cells, connections between DMV interior and cytosol could not be unambiguously identified, suggesting that dsRNA is compartmentalized by the DMV membranes. In addition, ET revealed a network of nucleocapsid protein-containing protein tubules, intertwined with the RVN. This potential intermediate in nucleocapsid formation, which was not observed in coronavirus-infected cells, suggests that arterivirus RNA synthesis and assembly are spatially coordinated.

### 3.3. Picornaviruses

Membrane remodeling in picornavirus-infected cells has been studied for more than 50 years. Massive virus-induced membrane modifications have been reported already in 1958 [70], but the origin of these membranes is still a matter of controversy. Several lines of evidence, including biochemical and structural data, suggest that the ER must play a major role in the formation of those structures [71,72,73]. Early in infection membranous replication factories contain markers of the Golgi [74,75], whereas markers of the ER, Golgi and lysosomes were all found to be associated with poliovirus replication sites late in infection [76]. Initial reports identified membrane rearrangements as U-bodies because of their horseshoe-like shape [70]. Later, Bienz *et al.* described rearranged membranes as clusters of single-membrane vesicles [71,77], while other reports [76,78] noticed the double-membrane morphology of picornavirus-induced vesicles. The single- *versus* double-membrane morphology of the vesicles was first interpreted as two different models of their formation. However, several recent publications suggest that picornavirus-induced membrane rearrangements might occur in a consecutive manner. Thus, early in poliovirus infection small clusters of single-membrane vesicles predominate that are transformed into bigger irregularly shaped single-membrane structures (Figure 3G) and, late in infection, replaced by either round or irregularly shaped DMVs [79]. Interestingly, the small clusters of single-membrane vesicles of ~100–200 nm diameter contain GM130, a *cis*-Golgi marker, but did not stain positive for calnexin, an ER marker. However, this does not exclude a role of the ER for biogenesis of these vesicles, because ER-resident proteins might be sorted out as these membranes are transformed. Although dsRNA and metabolically labeled viral RNA were detected in single-membrane vesicles and DMVs, the exponential phase of viral RNA synthesis correlates with the appearance of single-membrane and intermediate structures [79] arguing that these structures are most relevant for high level poliovirus RNA synthesis.

Similar results have been obtained with another member of the family *Picornaviridae*, coxsackie B3 virus (CVB3) that also induces the formation of single- and double-membrane compartments, whose relative abundance correlates with the stage of the replication cycle [80] (Figure 3F). Based on the observation that the Golgi apparatus disappears in CVB3-infected cells, the membrane rearrangements might originate from this organelle (Montserrat Bárcena, personal communication). Similar to poliovirus, single-membrane tubular clusters occur predominantly early in infection, whereas the number of DMVs increases as infection progresses. A budding event could account for the formation of the tubules, depicting an average length of 654 ± 291 nm and an average diameter of 81 ± 7 nm. A subsequent enwrapping of these single-membrane tubules via an “autophagy-like” mechanism could then lead to the formation of DMVs that have an average diameter of 159 nm ± 47 nm. This transformation may require several steps: (i) membrane pairing; (ii) induction of curvature; and (iii) membrane fusion [80]. This scenario would be consistent with the membrane surface of DMVs as an average-sized DMV with a diameter of 160 nm would be equivalent to a tubule with a length of 632 nm and a diameter of 81 nm. ER membranes were found near DMV clusters. However, in contrast to previous observations in nidovirus-infected cells, these DMVs were not connected to neighboring structures. In addition to these compartments, a third type of modification was detected in CVB3-infected cells: multilamellar structures, which are typical for the late phase of infection and that correspond to enwrapped DMVs: despite their various shapes and degrees of complexity, in all instances they contained one DMV, surrounded by one or several layers of curved cisternae.

In conclusion, these results, and similar observations made for Foot and Mouth Disease Virus (FMDV) (genus *Aphthovirus*) [81] suggest that members of the *Picornaviridae* family induce single- and double-membrane vesicles. They appear in a time-dependent manner and seem to evolve from each other, possibly in coordination with the progression of the viral replication cycle [80]. These membrane rearrangements occur independently from the used virus strain and cell line.

### 3.4. Togaviruses

Rubella Virus (RUBV) is an important human teratogenic virus and the only member of the genus *Rubivirus* (family *Togaviridae*) [82]. RUBV anchors its RNA synthesis machinery to membranes of a cell organelle known as “cytopathic vacuoles” (CPVs) that is derived from modified endosomes or lysosomes and has an average diameter of 600–2000 nm [83,84,85]. Freeze-fracture and ET analysis of RUBV-infected cells revealed a high complexity of CPVs that are composed of stacked membranes, rigid sheets, small vesicles and large vacuoles (Figure 3I) [86]. The CPVs are interconnected and linked to the endocytic pathway, as deduced from labeling experiments with endocytosed BSA-gold. Furthermore, rER cisternae, mitochondria and Golgi stacks are recruited around CPVs to build up RUBV factories. CPVs have several contacts with cellular organelles: they are coupled to the rER through protein bridges of ~10–15 nm and closely apposed membranes and they are attached to Golgi vesicles, whereas contacts with mitochondria were not detected [86]. It has been proposed that RNA synthesis occurs on vesicular membranes within the CPVs, which are linked to the cytosol and that the viral replicase molecules are associated with vesicles that transform with time into large vacuoles and straight elements [86]. This is supported by immunogold labeling revealing replicase components and dsRNA within the CPVs [83,84,87].

The modification of late endosomes and lysosomes is a feature that RUBV shares with alphaviruses, the other genus of the family *Togaviridae* [88,89]. Cells infected with alphaviruses like Semliki Forest Virus (SFV), Sindbis Virus and Western Equine Encephalitis Virus (WEEV) contain large CPVs with a diameter ranging between 600 and 2000 nm. The inner surface of these CPVs is covered with small invaginations or spherules that originate at the plasma membrane [90,91,92] (Figure 3J). These spherules are comprised of a single membrane forming a vesicle with a diameter of ~50 nm. In addition, the spherules were shown to be the site of viral RNA synthesis as deduced from metabolic labeling and detection by EM [89,91,92]. Importantly, the inside of the spherule is connected to the cytoplasm by a pore with a diameter of 5–10 nm. The spherules are formed at the plasma membrane by the concerted action of the viral nonstructural proteins (nsP1–nsP4) and genomic viral RNA [89]. Furthermore, Froshauer *et al.* [88] showed that the CPVs that contain the spherules possess endosomal and lysosomal markers.

Time course studies revealed that the spherules of SFV undergo an unprecedented large-scale movement between cellular compartments [93]. The spherules first form as blebs (exvaginations) at the plasma membrane. Then, they are internalized by an endocytic process requiring a functional actin-myosin network. The spherules therefore represent an unusual type of endocytic cargo. After endocytosis, spherule-containing vesicles, namely CPVs-I fuse with acidic endosomes and move along microtubules. This leads to the formation of a very stable compartment, where the spherules accumulate as invaginations on the outer surface of unusually large, acidic vacuoles localized in the pericentriolar region [93].

### 3.5. Caliciviruses

Members of the genus *Norovirus* (NoVs, family *Caliciviridae*) are major agents of acute gastroenteritis [94]. Ultrastructural examination of Murine Norovirus 1 (MNV-1)-infected cells revealed a striking change in their overall morphology and intracellular organization [95]. Structures resembling virus particles were observed within or next to single- or double-membrane vesicles in the cytoplasm. The vesiculated areas increase in size with time and by 24 hpi, large numbers of these vesicles and viral particles occupy most of the cytoplasm and displace the nucleus (Figure 3H). In addition, a complete rearrangement of the ER and loss of intact Golgi apparatus was observed. Both dsRNA and MNV-1 nonstructural protein 7, the RNA dependent RNA polymerase, localize to the limiting membrane of individual vesicle clusters by immuno-EM [96]. Immunofluorescence-based double-labeling showed that MNV-1 appears to recruit membranes derived from multiple cellular organelles and/or compartments: the ER, *trans*-Golgi apparatus and endosomes. However, despite extensive efforts, human norovirus cannot be grown in cultured cells [97]. Thus, detailed studies have not been possible, but it is assumed that replication structures are similar to those of MNV-1.

Feline Calicivirus (FCV), a member of the genus *Vesivirus* within this family, is a major agent of respiratory disease in cats, which replication originates also membranous rearrangements and vesicles [98].

### 3.6. Plant Viruses

Brome Mosaic Virus (BMV, family *Bromoviridae*) generates its replication factory by hijacking ER membranes, similar to what has been described for other plant viruses like Tobacco Mosaic Virus (TMV, family *Virgaviridae*) [99], Tobacco Echt Virus (TEV, family *Potyviridae*) [100] and Red Clover Necrosis Mosaic Virus (family *Tombusviridae*) [101]. However, other plant viruses such as Alfalfa Mosaic Virus (AMV) [102] and Cucumber Mosaic Virus (CMV), both belonging to the family *Bromoviridae*, and Turnip Yellow Mosaic Virus (TYMV, family *Tymoviridae*) [103] anchor their replication sites on chloroplasts. Cucumber Necrosis Virus (CNV), family *Tombusviridae*, utilizes peroxisomal membranes as replication platforms [104], while other plant viruses replicate on the surface of mitochondria [105].

Although the 3D architecture of these membranous replication sites remains largely unknown, their characteristics are strikingly similar to those for FHV (family *Nodaviridae*). Best studied is BMV that induces spherules, of similar size as the insect nodavirus FHV, in the ER close to the nucleus, where viral RNA synthesis and viral replication proteins are localized [106,107,108]. In the case of Beet Yellows Closterovirus (BYV, family *Closteroviridae*), TEM of infected plant cells revealed the formation of ~100 nm-diameter DMVs and multivesicular complexes (single-membrane vesicles surrounded by a common membrane) (Figure 3E) [109]. These multivesicular complexes often reside next to stacks of aligned filamentous BYV particles [110,111] and resemble the DMVs and VPs produced by nidoviruses and flaviviruses. Several BYV replication-associated proteins (L-PCP, MTR and HEL) colocalize with DMV and VP membranes, supporting the role of these structures as replications platforms [112,113]. The membranes in closterovirus DMVs and VPs are likely to be derived from ER for members of the genus *Crinivirus* [114] or mitochondria in case of *Ampelovirus* [115,116]. Whether these structures are “closed” or “necked” remain unknown.

## 4. Similarities and Differences between the Replication Factories of Flaviviruses and other (+) Strand RNA Viruses

Based on amino acid sequence homologies of their RNA-dependent RNA polymerases, (+) RNA viruses have been classified into three large supergroups [113,117,118]: supergroup I (*Picornavirata* or picorna-like group), including picorna-, corona-, arteri- and nodaviruses; supergroup II (*Flavivirata* or the flavi-like group), including tombus-, diantho-, pesti-, hepaci- and flaviviruses as well as single-strand RNA bacteriophages; supergroup III (*Rubivirata* or the alpha-like group), including tobamo-, hordei-, alpha- and rubiviruses as well as hepatitis E virus (HEV). These higher-order taxonomic units encompass diverse viruses infecting different hosts from almost all kingdoms of life. As discussed earlier [119], amongst these viruses two main architectures of remodeled membranes (morphotypes) can be found that may reflect two alternative strategies to induce the membranous microenvironments required to allow virus replication (summarized in Table 1).

The first morphotype involves the formation of negatively curved membranes, initiated by invaginations of the pre-existing membrane bilayer and giving rise to spherules, vesicles or vacuoles towards the lumen of the targeted cell organelle. These structures have been identified in a broad range of mammalian, plant and insect cells infected with viruses belonging to supergroups II and III. The second strategy involves the formation of membranes with positive curvature, giving rise to double-membrane structures that are the predominant characteristic of the replication factories of the picorna-like virus supergroup. The conservation of these two sorts of morphotypes in distantly related viruses supports the assumption of an evolutionary conserved mechanism. A striking finding in this regard was the observation that HCV, despite belonging to the flavi-like supergroup, induces DMVs whereas flaviviruses induce the formation of negatively curved membranes. To our current knowledge, HCV is the only member of the family *Flaviviridae* inducing the formation of membrane structures with positive curvature, suggesting that HCV might share common host cell pathways to induce membranous replication compartments with distantly related viruses such as corona-, arteri-, picorna-, calivi- or closteroviruses (Table 1). However, it still remains to be elucidated whether other members of the family *Flaviviridae*, belonging to the genera *Pestivirus* and *Pegivirus*, also utilize a picorna-like membrane remodeling strategy.

**Table 1:** Main structures induced by different (+) strand RNA viruses.

Morphotype	Virus Group and Representative Member		Name of Membrane Alteration	Size/Diameter	Membrane Source
Single-membrane vesicles	Nodaviruses	FHV	Spherules	~50 nm	Mitochondria
	Bromoviruses	BMV		~60 nm	ER (adjacent to the nucleus)
	Togaviruses	RUBV	Cytoplasmic vacuoles (CPVs)	600–2000 nm	Lysosomes
		SFV	CPVs with spherules	CPVs: 600–2000 nm Spherules: 50 nm	
	Flaviviruses	DENV	Invaginated vesicles	80–120 nm	ER
		WNV_KUN_		50–100 nm	
		TBEV		60–100 nm	
		MVEV, SLEV and JEV		75–145 nm	
		YFV		unknown	
Double-membrane vesicles	Hepaciviruses	HCV		~150 nm	ER
	Coronaviruses	SARS-CoV	Double membrane vesicles (DMVs)	200–300 nm	
		MHV		200–350 nm	
		MERS-CoV		150–320 nm	
		IBV		~200 nm	
	Arteriviruses	EAV		~95 nm	
	Picornaviruses	Poliovirus		100–300 nm	Golgi
		CV3B		~160 nm	
		FMDV		~100 nm	unknown (probably ER)
	Calicivirus	MNV-1		~200 nm	ER/Golgi
	Closterovirus	BYV		~100 nm	ER/Mitochondria

Abbreviations: FHV, Flock House Virus; BMV, Brome Mosaic Virus; RUBV, Rubella Virus; SFV, Semliki Forest Virus; DENV, Dengue Virus; WNV_KUN_, West Nile Virus (Kunjin strain); TBEV, Tick Borne Encephalitis Virus; MVEV, Murray Valley Encephalitis Virus; SLEV, St. Louis Encephalitis Virus; JEV, Japanese Encephalitis Virus; YFV, Yellow Fever Virus; HCV, Hepatitis C Virus; SARS-CoV, Severe Acute Respiratory Virus-Coronavirus; MHV, Mouse Hepatitis Virus; MERS-CoV (Middle East Respiratory Syndrome-Coronavirus); IBV, Infectious Bronchitis Virus; EAV, Equine Arterivirus; CVB3, Coxsackie B3 Virus; FMDV, Foot and Mouth Disease Virus; MNV-1, Murine Norovirus-1; BYV, Beet Yellows Virus.

A common feature associated with the spherule/vesicle/vacuole/-type of rearranged membranes is their size (50–150 nm diameter) and the presence of a pore connecting the interior of the vesicle with the cytoplasm [15,20,60,88]. Since RNA replication occurs in the vesicle interior, the pore allows exchange of nucleotides and RNA products with the cytoplasm. The size of the pore is variable, ranging from 4.4 nm in case of IBV-induced spherules to ~10 nm in case of membrane invaginations induced by flaviviruses. In contrast, in the majority of DMVs no such channel or pore has been detected. Nevertheless, as exemplified with nidoviruses, the inner compartments enclosed by interconnected DMVs contain the bulk of dsRNA [63], and in some cases they depict an electron-dense core assumed to correspond to viral RNA (IBV and EAV) [67,69]. Although this represents a functional enigma in terms of RNA synthesis and transport, the presence of dsRNA in the DMV interior does not necessarily indicate active RNA replication. Assuming a temporal regulation, it is possible that DMVs might be sites of RNA synthesis as long as they are linked to the cytoplasm, but replication would stop upon closure of the vesicles. Yet, another strategy appears to be used by enteroviruses, where active RNA replication has been detected on the cytosolic side of the membranous structures [71,120], consistent with the membrane topology of the nonstructural proteins catalyzing RNA replication [121,122,123]. As described above, studies conducted with picornaviruses revealed that the exponential phase of viral RNA synthesis coincides with the accumulation of single-membrane tubules [79,80]. Importantly, pulse-radiolabeling experiments localized sites of active RNA replication to the outer surface of single-membrane tubules [71] and isolation of the membranous replication factories and their subsequent visualization by EM revealed that they form rosette-like structures composed of virus-induced cytoplasmic vesicles [124]. RNA replication is thought to occur at sites where the vesicles cluster, whereas RNA translation probably takes place on the exposed periphery of the vesicles. This raises the question, what the role of DMVs in the replication cycle of picornaviruses might be. It is possible that DMVs either support RNA synthesis or serve as RNA storage sites (especially in case of closed DMVs). In this manner, DMVs might be involved in regulating viral RNA replication: by complete sealing of the viral replicase inside the vesicle, it would be inactive, thus regulating overall RNA copy number in the infected cell. Alternatively, DMVs might be an epiphenomenon, resulting from the over-expression of membrane-active proteins that accumulate especially during the late stages of infection.

The mechanism responsible for DMV formation is not clear. In case of picornaviruses, it is thought that single-membrane structures are the precursors of DMVs [79,80]. Nevertheless, DMV formation might also involve the autophagy machinery, or at least several components thereof, by a process analogous to the formation of autophagic vacuoles [76]. This hypothesis is supported by the morphological resemblance of DMVs and autophagosomes. It has been shown that the inhibition or stimulation of autophagy results in a modest inhibition or stimulation of poliovirus and coxsackie B3 virus yield, respectively, and there are also data supporting the involvement of autophagy in the replication of rhinovirus 2 and 14 [125,126]. However, Brabec-Zaruba *et al*. [127] reported that replication of rhinovirus 2 was insensitive to pharmacological manipulation of autophagy and did not induce detectable modification of LC3. This discrepancy might be due to the use of different cell types and experimental conditions.

The mechanism of DMV formation in case of HCV and coronaviruses is also unclear. Biochemical analysis of isolated host cell membranes associated with HCV RNA and proteins identified markers of the autophagy machinery, including LC3-II, the lipidated form of LC3 (LC3-II) that is generated upon activation of the autophagy machinery [46]. However, the role of autophagy in the HCV replication cycle is also a matter of controversy. For instance, immunolabeling did not identify LC3-II at those sites where nonstructural proteins accumulate [48]. Moreover, different roles of autophagy for the HCV replication cycle have been proposed. These include a role of autophagy in HCV RNA translation [128], initiation of RNA replication [129,130], production of infectious virus particles [131] or suppression of the innate antiviral defense [132,133]. To clarify these discrepancies, future studies should combine biochemical and cell biological approaches with ultrastructural analyses.

The autophagy machinery might also be involved in the formation of virus-induced membrane invaginations/spherules. For instance, Lee and coworkers provided evidence that DENV infection enhances autophagolysosome formation and that inhibition of the autophagy machinery by 3-methyladenine (3-MA) reduces DENV particle production [134]. However, the effects were moderate, arguing that this pathway may contribute to DENV replication to only a minor extent. Moreover, autophagy includes membrane wrapping, leading to double-membrane compartments involved in lysosomal degradation whereas DENV-induced vesicles are invaginations. Finally, immunolabeling experiments failed to detect lamp-1 at these vesicles [15], arguing against the involvement of lysosomes in the formation of the DENV replication vesicles. It remains to be determined whether autophagy is actively induced by these viruses to provide a compartment favoring replication or induced as a bystander defense against infection leading to degradation of the replicase proteins [135].

Another membrane compartment frequently induced by (+) RNA viruses are convoluted membranes (CMs) that were observed e.g., in SARS-CoV-, MHV-, WNV- or DENV-infected cells [13,15,18,63,65,66,136]. Morphologically, CMs resemble smooth ER membranes, lack ribosomes and in case of DENV are induced by the sole expression of NS4A [137,138]. CMs are often associated with late stages of infection, suggesting that DMV formation might precede the development of CMs. In SARS-CoV-infected cells, DMVs appear to be connected with CMs [63], while in MHV-infected cells no such connections have been observed [65]. The role of CMs for the viral replication cycle is not well understood. In case of WNV_KUN_, CMs are supposed to be the site of polyprotein processing [18,136]. This conclusion is based primarily on the strong immunolabeling for NS2B and NS3 and the absence of NS1 and NS4B. Since polyprotein cleavage occurs co-translationally and thus, should happen at the rER, this model would require the formation of rather stable processing intermediates that are transferred from the rER to the CMs where further cleavage would occur. Alternatively, at least in case of DENV, CMs might represent a storage site for proteins and lipids involved in viral replication that can be recruited to vesicles upon demand. The fact that CMs are physically linked with ER-containing invaginations and contain NS3 would be consistent with this assumption [15]. Along these lines, the fact that insects are cholesterol auxotrophs and lack several enzymes in the cholesterol biosynthesis pathway [139], suggests that cholesterol might be a key component of CM structures, which would explain their absence in infected insect cells [16].

## 5. Role of Viral Proteins in the Formation of the Replication Organelles

Viral replication complexes are targeted to the respective membranous organelle primarily by nonstructural (NS) proteins rather than viral RNA [140]. These NS proteins seem to have some specificity in recognizing organelle subpopulations and often contain multiple hydrophobic domains implicated in membrane targeting and rearrangement. The molecular mechanisms orchestrating the formation of these complex structures are still poorly understood, but it is clear that NS proteins, often working in a concerted action, are key players in replication factory biogenesis.

### 5.1. Single-Membrane Vesicle Inducers

One well-studied example among the single-membrane vesicle inducers is BMV where it was shown that the sole expression of the NS protein 1a is sufficient to induce the formation of single-membrane spherules resembling the ones observed in infected cells. These spherules had a diameter of 50–70 nm, resided in the ER lumen and were shown to be the site of viral RNA synthesis [108]. In case of the insect nodavirus FHV, protein A and replication competent RNA were required for induction of the spherules. Expression of protein A alone induced only “zippering” of the surfaces of adjacent mitochondria, but did not induce spherules. Thus, protein A is necessary, but not sufficient for spherule formation. Moreover, spherules were not formed when replication-competent FHV RNA templates were expressed with a protein A mutant lacking polymerase activity or when wild-type protein A was expressed with a replication-incompetent FHV RNA template. Thus, the membranous FHV replication compartment requires both a viral protein and active RNA synthesis [141].

Feline Calicivirus (FCV) infection results in rearrangement of intracellular membranes and production of numerous membrane-bound vesicular structures on which viral genome replication is thought to occur. Expression of individual FCV nonstructural proteins revealed that p30 induces significant reorganization of the ER into large, fenestrated membrane networks, resembling the structures found in infected cells [142]. Moreover, expression of p39 and p32, two additional FCV NS proteins, induced extensive reorganization of the ER and the nuclear envelope suggesting that the ER is the primary source of the membranous replication factory [142].

Flavivirus membrane rearrangements are mainly induced by NS4A, as suggested by recent studies with WNV_KUN_ and DENV [137,138], but it is unknown whether the same applies to NS4A of TBEV. In case of DENV, NS4A is thought to contain a central peripheral membrane domain that intercalates into the luminal leaflet of the ER membrane [138]. It is tempting to speculate that NS4A oligomers [143] might dilate the luminal leaflet, resulting in membrane invaginations towards the ER lumen. However, the sole expression of NS4A is not sufficient to induce ER membrane invaginations that have been detected in infected cells. Instead, expression of NS4A lacking the C-terminal 2K fragment (corresponding to fully processed NS4A) induced ER membrane rearrangements reminiscent of CMs, whereas unprocessed NS4A/2K did not induce membrane alterations [138]. These results provide strong evidence that processing at the NS4A-2K site is required for the induction of membrane alterations. The critical role of polyprotein cleavage for induction of membrane rearrangements is supported by studies conducted with WNV_KUN_. There it was shown that a regulated cleavage of a NS4A/2K/4B precursor by the viral NS2B/3 protease is needed for induction of membrane rearrangements [137]. However, the same study reported that expression of full-length (uncleaved) WNV_KUN_ NS4A/2K led to membrane alterations similar to those induced in infected cells whereas the 2K fragment impaired the ability of NS4A to induce membrane rearrangements. Whether this reflects a biological difference between WNV_KUN_ and DENV NS4A or is due to the use of alternative experimental approaches remains to be determined.

One of the most fascinating mechanisms employed by (+) RNA viruses to induce their replication factories is used by SFV. It was shown that the spherules of SFV arise by blebbing at the surface of the plasma membrane [93]. These blebs are internalized and after fusion with lysosomes give rise to large cytoplasmic vacuoles. Formation of these membrane alterations requires the viral protein nsP1, which has several functions. It has guanine-7-methyltransferase and guanylyltransferase activities and thus is critically involved in capping of the viral RNAs [144,145,146], but at the same time has affinity to lipids [89]. In fact, of the four NS proteins of SFV, only nsP1 has affinity for membranes, and when expressed alone, it is specifically targeted to the inner surface of the plasma membrane [147]. NsP1 is a monotopic membrane protein and its affinity for membranes is dictated by an amphipathic α-helix, located in the central region of the protein [148,149]. NsP1 has a specific affinity for negatively charged phospholipids, which might account for its prevalent localization to the plasma membrane, where such lipids are enriched. Membrane binding of nsP1 via its amphipathic α-helix is essential for alphavirus replication [93]. However, nsP1 is not sufficient for cytoplasmic vacuole formation. For instance, it was found that nsP3 contributes to the transport of the replicase polyprotein from the plasma membrane to the surface of endosomes [150]. These results indicate that nsp1 has to cooperate with other viral and cellular factors to allow formation of the cytoplasmic vacuoles. Furthermore, in a recent study Kallio *et al*. [151] have shown that the size of the spherule is dependent on the length of the RNA template, in contrast to what has been observed for FHV, another spherule-inducer [141]. These results indicate that in addition to the NS proteins, the viral RNA template itself critically determines the morphology of the membranous vesicles.

### 5.2. Double-Membrane Vesicle Inducers

In order to induce the variety of membrane alterations observed in CVB3 -infected cells (Figure 3F), several membrane-remodeling mechanisms are required: induction of membrane curvature, membrane fusion and membrane-membrane interactions [80]. These rearrangements require the enteroviral proteins 2BC and 3A; their coexpression generates ER membrane-derived structures mimicking those observed during viral infection [78]. Importantly, 2B and 2C both contain an amphipathic α-helix [152,153,154], a well-known curvature-inducing motif [155]. Along the same lines, FMDV 2B and 2BC locate to the ER when expressed on their own and cause a swelling of ER cisternae [156].

In case of HCV, we recently found that a concerted action of NS3/4A, NS4B, NS5A and NS5B is required to generate the membranous web. Furthermore, all these replicase components were capable of inducing membrane vesiculation with NS5A having the highest potential to trigger membrane curvature. Importantly, some of these NS5A-induced structures corresponded to DMVs [48]. In addition, NS4B also plays an important role in triggering rearrangements of intracellular membranes [45]. NS4B is an integral membrane protein containing two N-terminal amphipathic α-helices, a highly hydrophobic central core domain composed of four putative transmembrane segments, and a highly conserved C-terminal domain that is thought to harbor two α-helices (reviewed in [157]). A recent study has demonstrated that NS4B oligomerizes through multiple conserved determinants and that oligomerization appears to be required for membranous web induction [158]. Indeed, mutations affecting the highly conserved C-terminal domain impairing NS4B self-interaction resulted in the formation of aberrant DMVs arguing for a central role of NS4B in formation of functional replication compartments [159].

Studies on arteriviruses revealed that the sole expression of EAV nsp2 and nsp3 is sufficient to induce membrane structures similar to those generated during EAV infection [160]. Mutations within nsp3, which is a tetra-spanning integral membrane protein, alter membrane rearrangements, highlighting the importance of this protein for the biogenesis of EAV-induced DMVs [161]. In case of SARS-CoV, nsp3, nsp4 and nsp6 were found to be sufficient to induce the formation of DMVs that are similar to those observed in SARS-CoV-infected cells [162]. These DMVs were, however, smaller in diameter, suggesting a role for other viral proteins or the presence of viral RNA in determining the DMV morphology. Importantly, EM analysis of nsp4 mutants that are impaired in RNA replication and virus growth, revealed an aberrant morphology of DMVs as well as an increased prevalence of CMs [163]. Another important viral protein involved in inducing the SARS-CoV membranous replication factory is nsp6, which is predicted to contain seven transmembrane segments and a hydrophilic cytoplasmic domain [164]. Nsp6 was shown to induce vesicles containing Atg5 and LC3-II as well as phosphatidylinositol-3-phosphate, thus sharing many features with omegasomes, which are omega-shaped membrane compartments that are formed during activation of autophagy [164]. This result suggests that autophagy might contribute to the formation of the membranous replication site of SARS-CoV.

## 6. Conclusions and Future Perspectives

In the last couple of years, our knowledge of the architecture of the replication factories induced by (+) RNA viruses has increased substantially. This is primarily due to the more widespread use of ET and other high-resolution imaging methods. Nevertheless, our knowledge is still rather restricted to descriptions of the morphologies of these complex structures, whereas our understanding of their biogenesis in most cases is very rudimentary. More efforts are required to elucidate the role of the viral proteins in the formation of the replication vesicles, to identify the involved cellular components and the mechanisms used by these proteins to subvert and exploit cellular pathways to establish membranous replication factories. This includes determination of the 3D structure of involved (viral) proteins as well as evaluation of host cell factors and lipids contributing to biogenesis and activity of the replication compartment. In addition, further studies are needed to understand how viruses utilize these compartments to coordinate the different steps of their life cycle (replication, assembly and release) in space and time to achieve efficient replication.
